# 1537. Utilizing GPS Technology to Assess Effects of Activity Space Exposure to Violent Crimes and HIV Preexposure Prophylaxis (PrEP) Use among Sexual Minority Men

**DOI:** 10.1093/ofid/ofad500.1372

**Published:** 2023-11-27

**Authors:** John Flores, Seann Regan, Tyrone Moline, Byoungjun Kim, John Schneider, Dustin Duncan

**Affiliations:** University of Chicago Hospital, Chicago, Illinois; Columbia University, New York, New York; Columbia University, New York, New York; New York University Grossman School of Medicine, New York, New York; University of Chicago Medicine, Chicago, Illinois; Columbia University, New York, New York

## Abstract

**Background:**

Across the USA, young Black men who have sex with men (Black MSM) have the highest incidence of HIV of any demographic. HIV Preexposure Prophylaxis (PrEP) adherence has been shown to substantially reduce HIV acquisition. Exposure to violent crimes has been associated with poor medication adherence and adverse outcomes such as with cardiovascular and mental health, but there is limited data on exposure to violent crime and PrEP adherence. Our cohort study sought to utilize global positioning devices (GPS) to assess relationships between exposure to violent crime and PrEP use among Black MSM in the city of Chicago.

**Methods:**

Between January 2018 to December 2019 participants were recruited and asked to complete a digital survey instrument which assessed of their social networks & sexual health behaviors, including PrEP use. They were provided with a GPS device to be worn prospectively for two weeks (Figure 1). Crime related data was extracted through the Chicago Data Portal, and then geocoded and analyzed utilizing ArcGIS computing software (Figure 2). Statistical analyses with exposure to crime was run between those who currently use PrEP and those who no longer adherent to PrEP.

**Figure 1:**
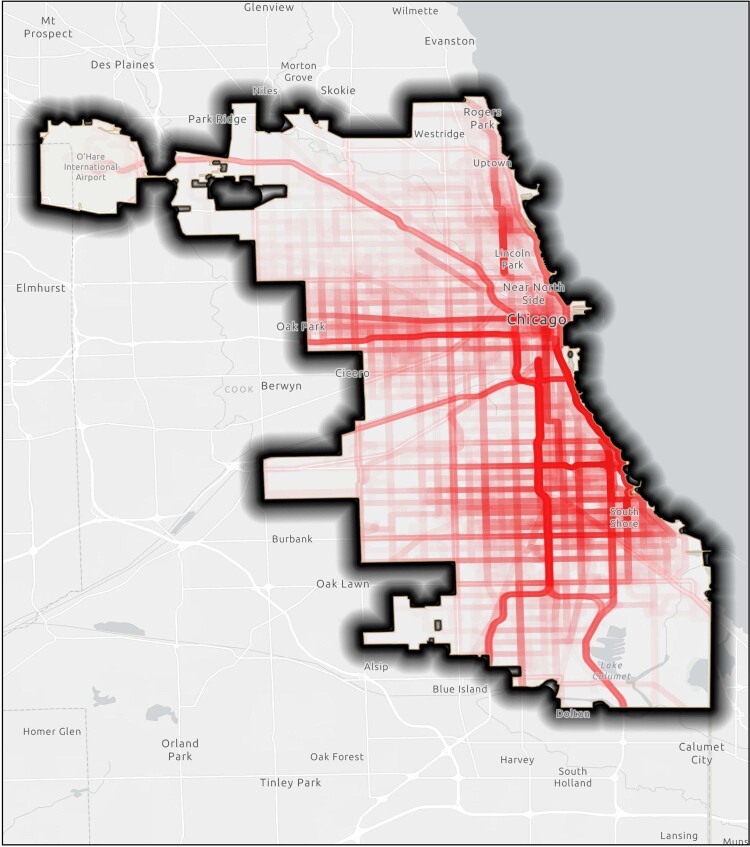
GPS Activity Space

Geocoded activity spaces of the research participants over two week period with 200 meter activity space buffer.

**Figure 2:**
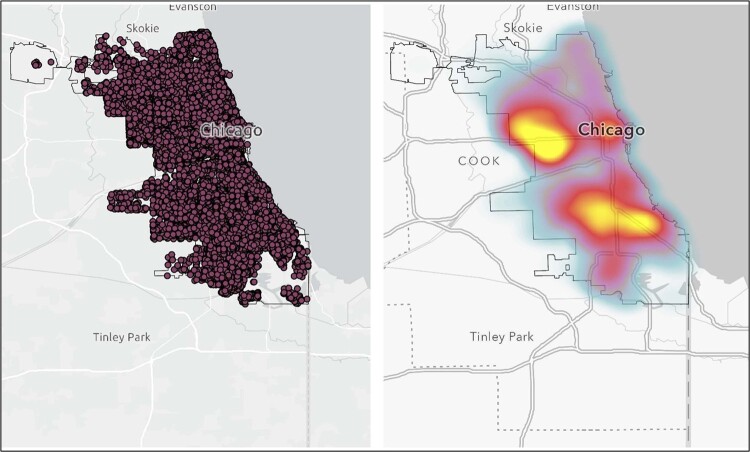
Violent Crime Point Data and Heat Map

This figure shows two maps with the first representing extracted point data of all police reported crimes from January 2018 - December 2019, and the second map showing hot spot analysis based off increasing density with yellow being highest density and blue lowest density.

**Results:**

317 HIV seronegative participants were recruited and wore GPS devices (Table 1). 193 reported a previous history of PrEP use and 85 reported current PrEP use. T-test comparison (Table 2) had GPS activity spaces among those who previously used PrEP had a statistically significant higher density of violent crime (p=0.02), and no difference with property crime density (p=0.46) or drug crime density (p-0.10). Logistic regression adjusted for covariates showed no statistically significant difference between each of the crime densities.

**Table 1:**
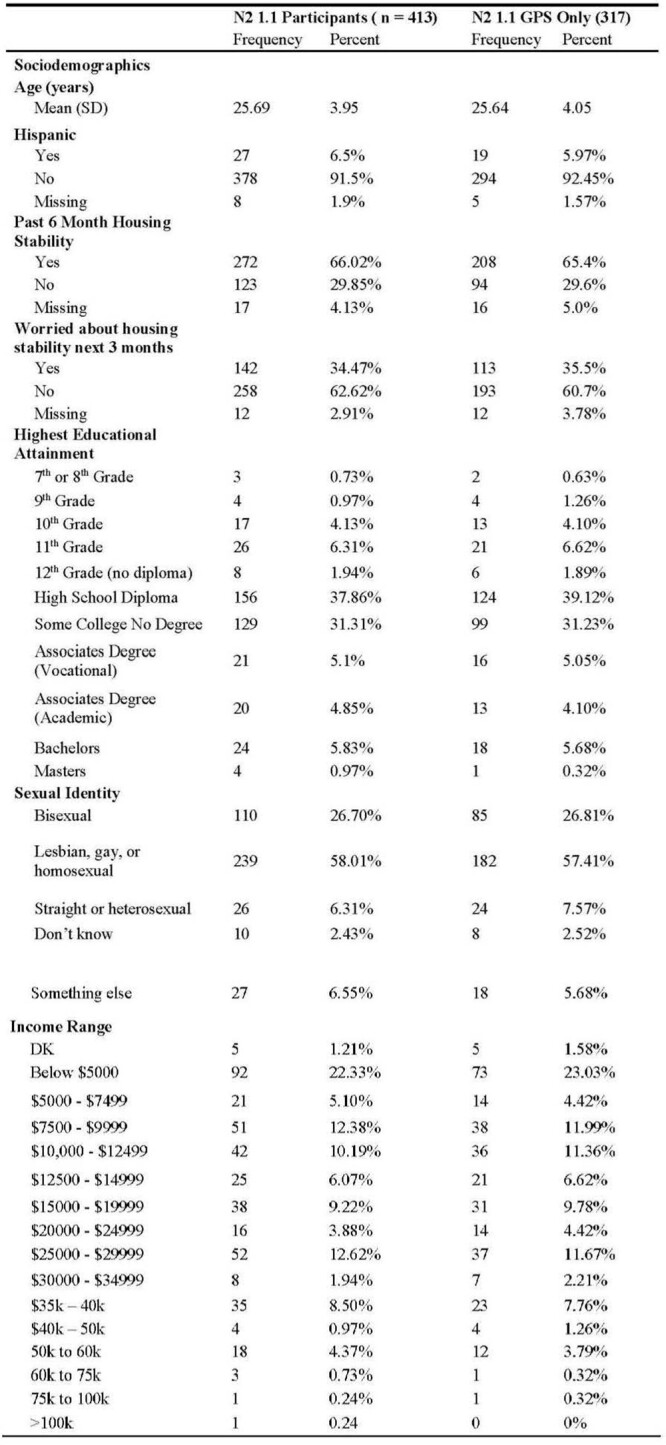
Descriptive Characteristics of Participants

**Figure:**
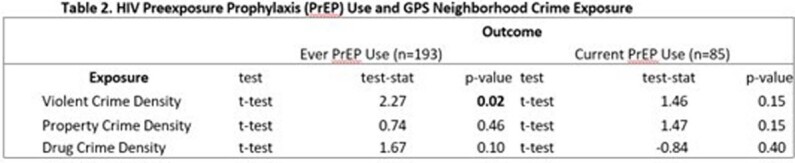

This table shows the t-test analysis comparing those not currently adherent to PrEP and those who are currently adherent with regards to GPS activity space exposure to violent, poverty, and drug related crimes.

**Figure:**
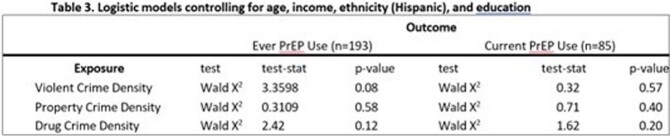

This table shows the logistical regression analysis comparing those not currently adherent to PrEP and those who are currently adherent with regards to GPS activity space exposure to violent, poverty, and drug related crimes adjusted for age, income, ethnicity (Hispanic), and education.

**Conclusion:**

Our GPS data show a significantly higher density of violent crime among the whole cohort for those with previous PrEP use than those with current. However, additional investigation is required to identify which adjusted variables explain the finding. Additionally, the GPS methods in our study also examined the multiple spatial contexts that an individual experiences, not just the residential neighborhood environment (Figure 3). This is significant in advancing in the field of HIV epidemiology and prevention.

**Figure 3:**
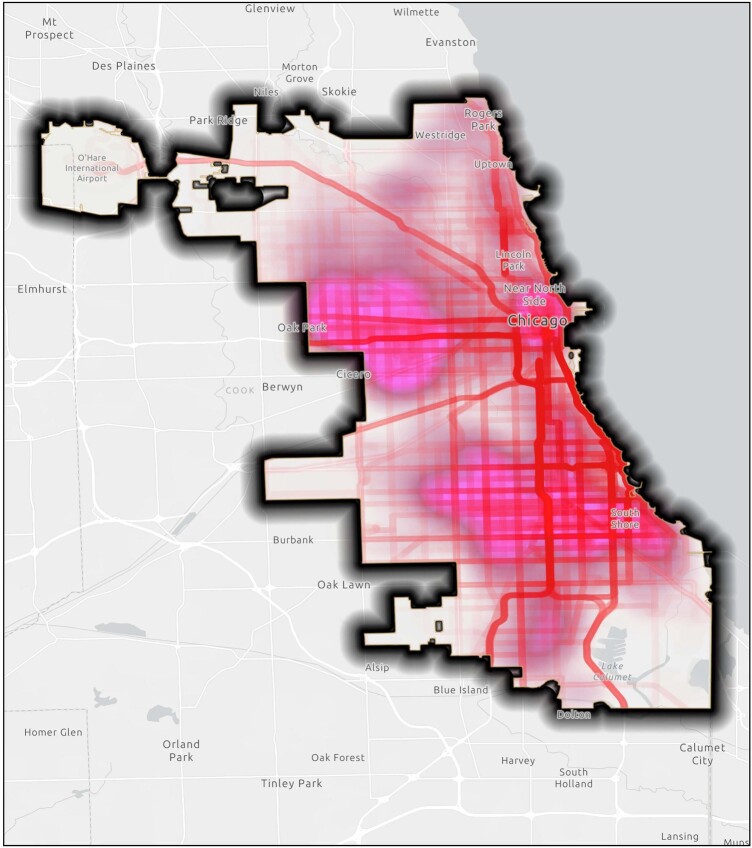
Activity Space & Violent Crime Density

This map shows the overlay of the concentrations of the activity spaces of the participants and the high concentrations of violent crime in the city of Chicago.

**Disclosures:**

**All Authors**: No reported disclosures

